# PDS5B inhibits cell proliferation, migration, and invasion via upregulation of LATS1 in lung cancer cells

**DOI:** 10.1038/s41420-021-00537-6

**Published:** 2021-06-21

**Authors:** Hui Xu, Wenjing Zhou, Fan Zhang, Linhui Wu, Juan Li, Tongtong Ma, Tong Cao, Chaoqun Lian, Jun Xia, Peter Wang, Jia Ma, Yuyun Li

**Affiliations:** 1 Department of Laboratory Medicine, School of Laboratory Medicine, Bengbu Medical College, Bengbu, Anhui 233030 China; 2 Bengbu Medical College Key Laboratory of Cancer Research and Clinical Laboratory Diagnosis, Bengbu Medical College, Bengbu, Anhui 233030 China; 3 Department of Pharmacology, Adagene Limited Company, Suzhou, Jiangsu 215000 China; 4 School of Laboratory Medicine, Bengbu Medical College, Bengbu, Anhui 233030 China; 5 Department of Clinical Laboratory, The First Affiliated Hospital of Bengbu Medical College, Bengbu, Anhui 233004 China; 6 Department of Biochemistry and Molecular Biology, School of Laboratory Medicine, Bengbu Medical College, Bengbu, Anhui 233030 China

**Keywords:** Non-small-cell lung cancer, Preclinical research

## Abstract

PDS5B (precocious dissociation of sisters 5B) plays a pivotal role in carcinogenesis and progression. However, the biological functions of PDS5B in lung cancer and its underlying mechanisms are not fully elucidated. In the present study, we used MTT assays, wound-healing assays, and transwell migration and invasion approach to examine the cell viability, migration, and invasion of non-small cell lung cancer (NSCLC) cells after PDS5B modulation. Moreover, we investigated the function of PDS5B overexpression in vivo. Furthermore, we detected the expression of PDS5B in tissue samples of lung cancer patients by immunohistochemical study. We found that upregulation of PDS5B repressed cell viability, migration, and invasion in NSCLC cells, whereas downregulation of PDS5B had the opposite effects. We also observed that PDS5B overexpression retarded tumor growth in nude mice. Notably, PDS5B positively regulated LATS1 expression in NSCLC cells. Strikingly, low expression of PDS5B was associated with lymph node metastasis in lung cancer patients. Our findings suggest that PDS5B might be a therapeutic target for lung cancer.

## Introduction

Lung cancer is the most common tumor and is the first leading cause of mortality in the United States [1]. It will be expected that 228,820 new patients with lung cancer and 135,720 deaths for this disease will be happened in the United States in 2020 [1]. Approximately 25% of all cancer deaths are because of lung cancer, and the 5-year relative survival rate of lung cancer is 19% [1]. Fifty-seven percent of patients with lung cancer have metastasis when they were diagnosed, leading to a 5% survival rate for these metastatic lung cancer patients [2]. The treatment strategies include surgery, chemotherapy, immunotherapy, and combination therapies [3–5]. Although the therapies have been improved, the outcomes of lung cancer patients are still not satisfactory. Therefore, it is necessary to discover new biomarkers for the prediction of early-stage lung cancer and to find novel targets for the treatment of lung cancer.

Evidence has demonstrated that several genes play an important role in lung cancer development and malignant progression. For example, the epidermal growth factor receptor often has mutations in lung adenocarcinoma [6]. Moreover, microRNAs, long non-coding RNA (lncRNAs), and circular RNAs (circRNAs) are critically involved in lung tumorigenesis [7–11]. Hippo signaling pathway has been identified to participate in lung tumor initiation and progression [12–14]. It has been known that Hippo pathway negatively controls the expression of YAP and TAZ, two transcriptional co-activators. This pathway consists of several kinases such as mammalian STE20-like protein kinase 1 (MST1), MST2, large tumor suppressor 1 (LATS1), and LATS2. MST1/2 activates LATS1/2 and MOB1A/1B via phosphorylation, and LATS1/2 activates YAP and TAZ phosphorylation, leading to inhibition of YAP and TAZ activation [15, 16].

Precocious dissociation of sisters 5B (PDS5B), also known as APRIN, was reported to participate in carcinogenesis. Knockdown of PDS5B disrupts stem cell programs by regulating Oct4, Nanog, and SOX2 patterns in embryonal carcinoma [17]. One study revealed that PDS5B expression is correlated with histological grade in breast cancer and the treatment efficacy of chemotherapy in breast cancer patients [18]. PDS5B, as a BRCA2-interacting protein, is required for DNA repair and genome integrity in breast cancer cells [18]. Another study demonstrated that miR-27a represses the expression of PDS5B in prostate cancer cells, leading to the promotion of cell viability [19]. PDS5B exhibits frameshift mutations in gastric cancer and colorectal cancer patients, which is associated with weak or negative PDS5B immunostaining [20]. In addition, a frameshift mutation of PDS5B is observed in breast cancer tissues and lymph nodes [21]. PDS5B is highly expressed in oral squamous cell carcinomas, but PDS5B is not correlated with the expression of proliferation cell markers such as p53 and ki-67 [22]. The functions and role of PDS5B in lung cancer cells have not been fully elucidated. Therefore, in this study, we explored the biological functions of PDS5B in lung cancer cells and determined its underlying molecular mechanism.

## Results

### PDS5B overexpression inhibits cell viability

**Fig. 1:**
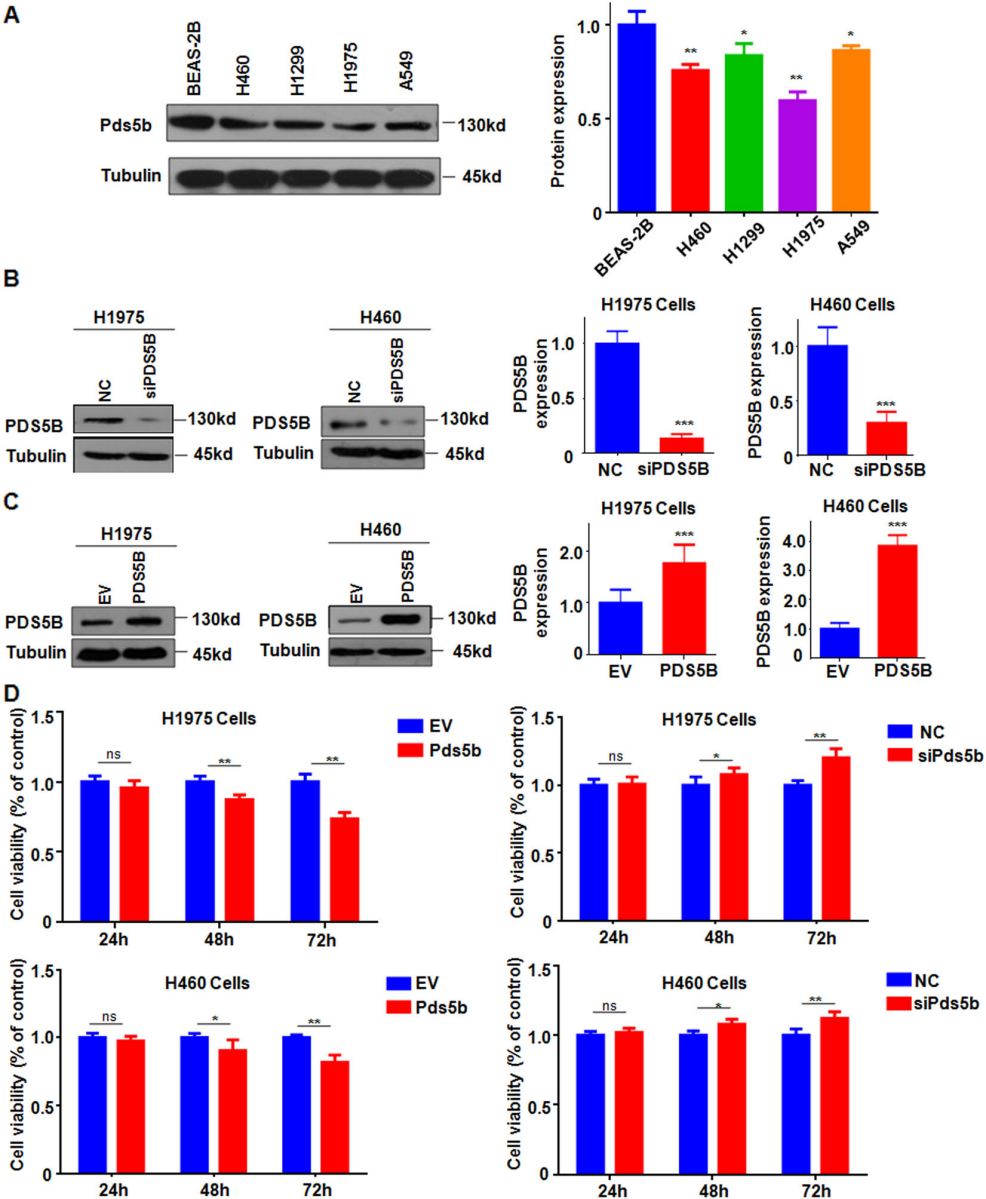
PDS5B overexpression represses cell viability. **A** Left panel: the expression of PDS5B was measured in the panel of lung cancer cells and the normal lung epithelial BEAS-2B cells. Right panel: Quantitative results of PDS5B expression. **P < 0.01 vs BEAS-2B cells. **B** Left panel: The expression of PDS5B was decreased in NSCLC cells after PDS5B siRNA transfection. Right panel: quantitative results of PDS5B expression. ***P < 0.001 vs NC group. *NC* nonspecific control siRNA, *siPDS5B* PDS5B siRNA. NC group is the control group. **C** Left panel: the expression of PDS5B was increased in NSCLC cells after PDS5B cDNA transfection. Right panel: quantitative results of PDS5B expression. ***P < 0.001 vs EV group. *EV* empty vector, *PDS5B* PDS5B cDNA. EV group is the control group. **D** Effects of PDS5B modulation on cell viability at different times in NSCLC cells were examined by MTT assays. **P < 0.01 vs control group. *NC* nonspecific control siRNA, *siPDS5B* PDS5B siRNA. *EV* empty vector, *PDS5B* PDS5B cDNA. All data are representative of three independent experiments. Data are shown as mean ± SD from experiments in triplicate.

The expression levels of PDS5B in the panel of lung cancer cells and the normal lung epithelial BEAS-2B cells were measured by western blotting. We found that PDS5B is highly expressed in BEAS-2B cells (Fig. 1A). To determine the role of PDS5B in lung cancer cells, PDS5B expression was downregulated by siRNA transfection in non-small cell lung cancer (NSCLC) cells. Our Western blotting data demonstrated that PDS5B expression was downregulated in both H1975 and H460 cell lines (Fig. 1B). Moreover, we also transfected PDS5B cDNA into NSCLC cells via Lipofectamine 3000. We observed that PDS5B expression was elevated in H1975 and H460 cells after cDNA transfection (Fig. 1C). Next, we explored whether PDS5B modulation affected the viability of NSCLC cells by MTT assays. The results from our MTT assays revealed that downregulation of PDS5B promoted cell viability, whereas overexpression of PDS5B repressed the viability of NSCLC cells (Fig. 1D).

### PDS5B overexpression suppresses cell migration and invasion

**Fig. 2:**
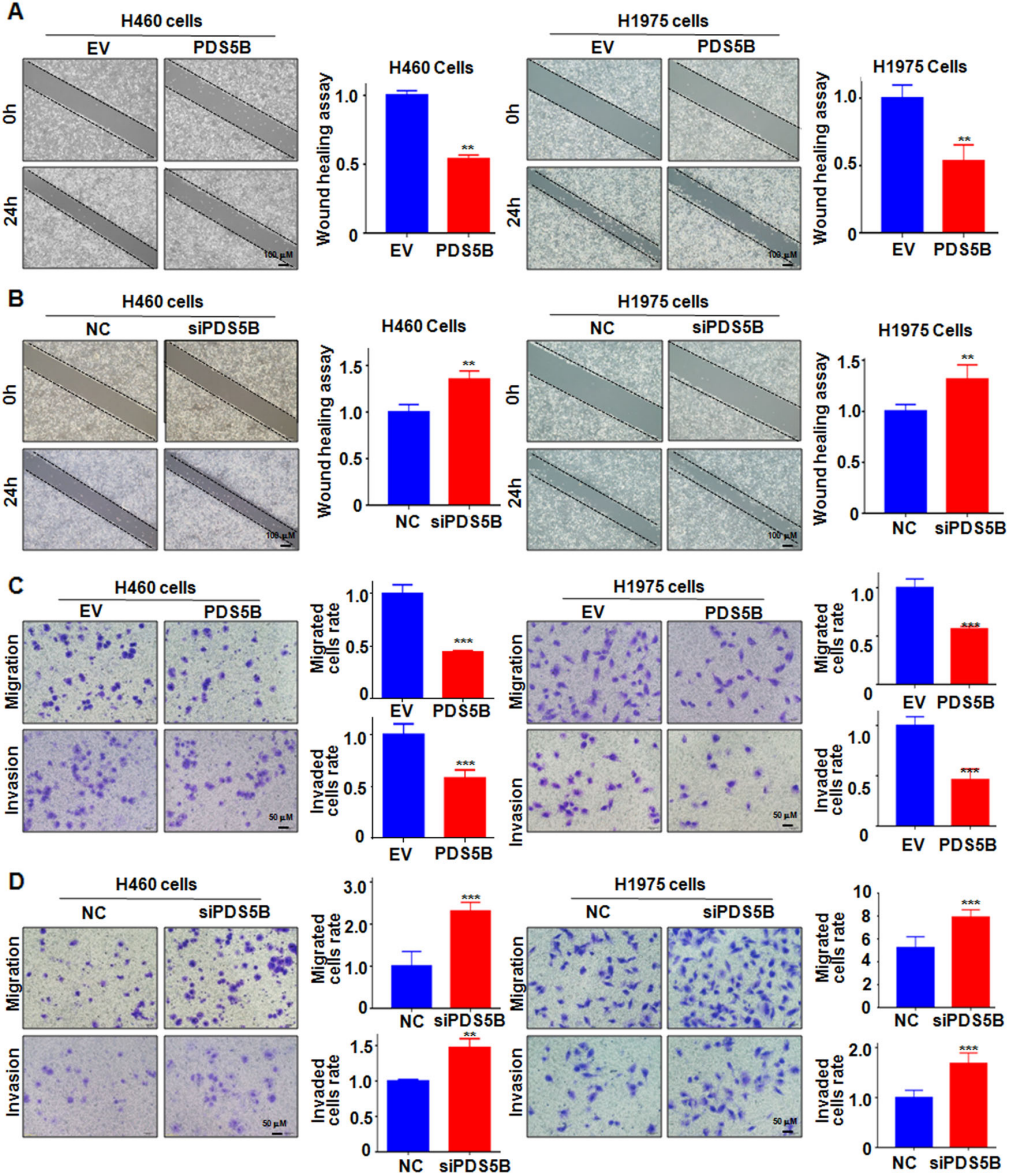
PDS5B overexpression retards cell motility. **A** Left panel: wound closure was examined by wound-healing assays in NSCLC cells after PDS5B cDNA transfection. Right panel: quantitative data of wound closure. **P < 0.01 vs EV group. *EV* empty vector, *PDS5B* PDS5B cDNA. EV group is the control group. **B** Left panel Wound closure was detected by wound-healing assays in NSCLC cells after PDS5B siRNA transfection. Right panel: quantitative data of wound closure. **P < 0.01 vs NC group. *NC* nonspecific control siRNA, *siPDS5B* PDS5B siRNA. NC group is the control group. **C** Left panel cell motility was examined in NSCLC cells after PDS5B cDNA transfection. Right panel: quantitative data of cell motility. ***P < 0.001 vs EV group. EV group is the control group. **D** Left panel: cell motility was measured in NSCLC cells after PDS5B siRNA transfection. Right panel: quantitative data of cell motility. **P < 0.01, ***P < 0.001 vs NC group. NC group is the control group. Data are shown as mean ± SD from experiments in triplicate.

To elucidate whether PDS5B could affect migration and invasion of lung cancer cells, wound-healing assays were performed in H1975 and H460 cells after PDS5B modulation. We found that overexpression of PDS5B delayed the wound closure in H1975 and H460 cells, indicating that PDS5B upregulation repressed cell migration (Fig. 2A). In line with this finding, downregulation of PDS5B promoted wound closure in NSCLC cells (Fig. 2B). To further validate the role of PDS5B in controlling migratory capacity, transwell chamber migration assays were conducted in NSCLC cells after PDS5B changes. Overexpression of PDS5B reduced the migrated cell numbers in H1975 and H460 cells (Fig. 2C). Moreover, transwell invasion assay data demonstrated that PDS5B overexpression decreased the invaded cell numbers through the membrane with coating Matrigel (Fig. 2C). Consistently, PDS5B knockdown promoted cell migratory and invasive capacities in NSCLC cells (Fig. 2C). Taken together, PDS5B governs the migration and invasion of NSCLC cells.

### PDS5B overexpression increases LATS1 expression

**Fig. 3:**
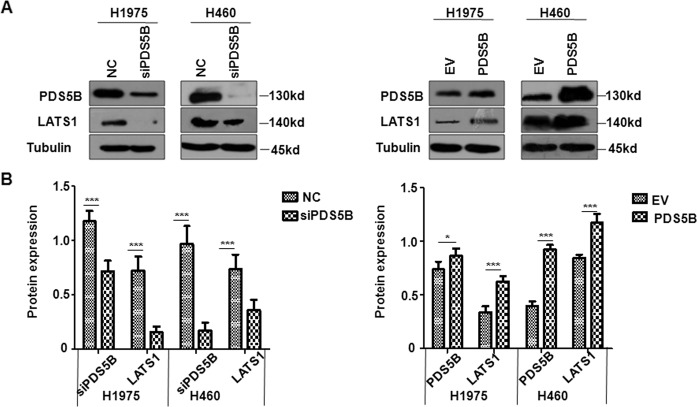
PDS5B regulates LATS1 expression. **A** The expression of PDS5B and LATS1 was examined by western blotting in H1975 and H460 cells after PDS5B modulation. *EV* empty vector, *PDS5B* PDS5B cDNA. *NC* nonspecific control siRNA, *siPDS5B* PDS5B siRNA. NC and EV groups are the control groups. **B** Quantitative data of western blotting results. *P < 0.05, ***P < 0.001 vs control group. Data are shown as mean ± SD from experiments in triplicate.

To determine the underlying mechanism of PDS5B-mediated antitumor activity in NSCLC cells, we measured the expression of LATS1 in H1975 and H460 cells after PDS5B modulation by western blotting analysis. Our data uncovered that PDS5B upregulation elevated the expression of LATS1 in H1975 and H460 cells, whereas PDS5B silencing alleviated the LATS1 expression levels in NSCLC cells (Figs. 3A, B). These results suggest that PDS5B positively regulates the LATS1 expression.

### Overexpression of LATS1 rescues PDS5B knockdown-induced cell viability

**Fig. 4:**
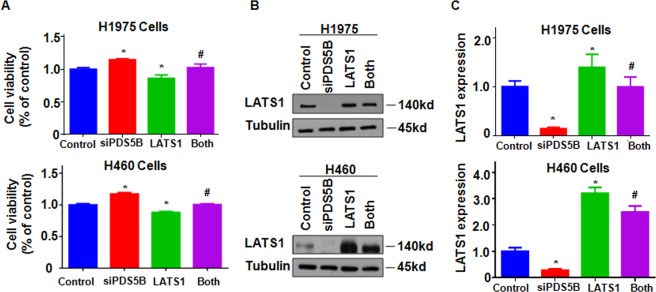
Overexpression of LATS1 rescues PDS5B knockdown-induced cell viability. **A** Cell viability was determined by MTT assays in NSCLC cells after co-transfection of LATS1 cDNA and PDS5B siRNA. **P < 0.05 vs control group; ^#^*P* < 0.05 vs PDS5B siRNA alone or LATS1 cDNA alone. *Control* nonspecific control siRNA and empty vector, *siPDS5B* PDS5B siRNA, *LATS1* LATS1 cDNA, Both: siPDS5B + LATS1. Data are shown as mean ± SD from experiments in triplicate. **B** The expression of LATS1 was examined in NSCLC cells after co-transfection of LATS1 cDNA and PDS5B siRNA. **C** Quantitative data of LATS1 expression. Data are shown as mean ± SD from experiments in triplicate.

To dissect whether PDS5B could govern cell viability via regulating LATS1 pathway, we overexpressed LATS1 in NSCLC cells after PDS5B downregulation. Overexpression of LATS1 reduced the viability of H1975 and H460 cells (Fig. 4A). Importantly, LATS1 overexpression abolished the promotion of cell viability that was induced by the knockdown of PDS5B in both NSCLC cell lines (Fig. 4A). Western blotting analysis data showed that upregulation of LATS1 abrogated the PDS5B knockdown-mediated inhibition of LATS1 in NSCLC cells (Figs. 4B, C).

### LATS1 overexpression abrogates PDS5B knockdown-mediated motility

**Fig. 5:**
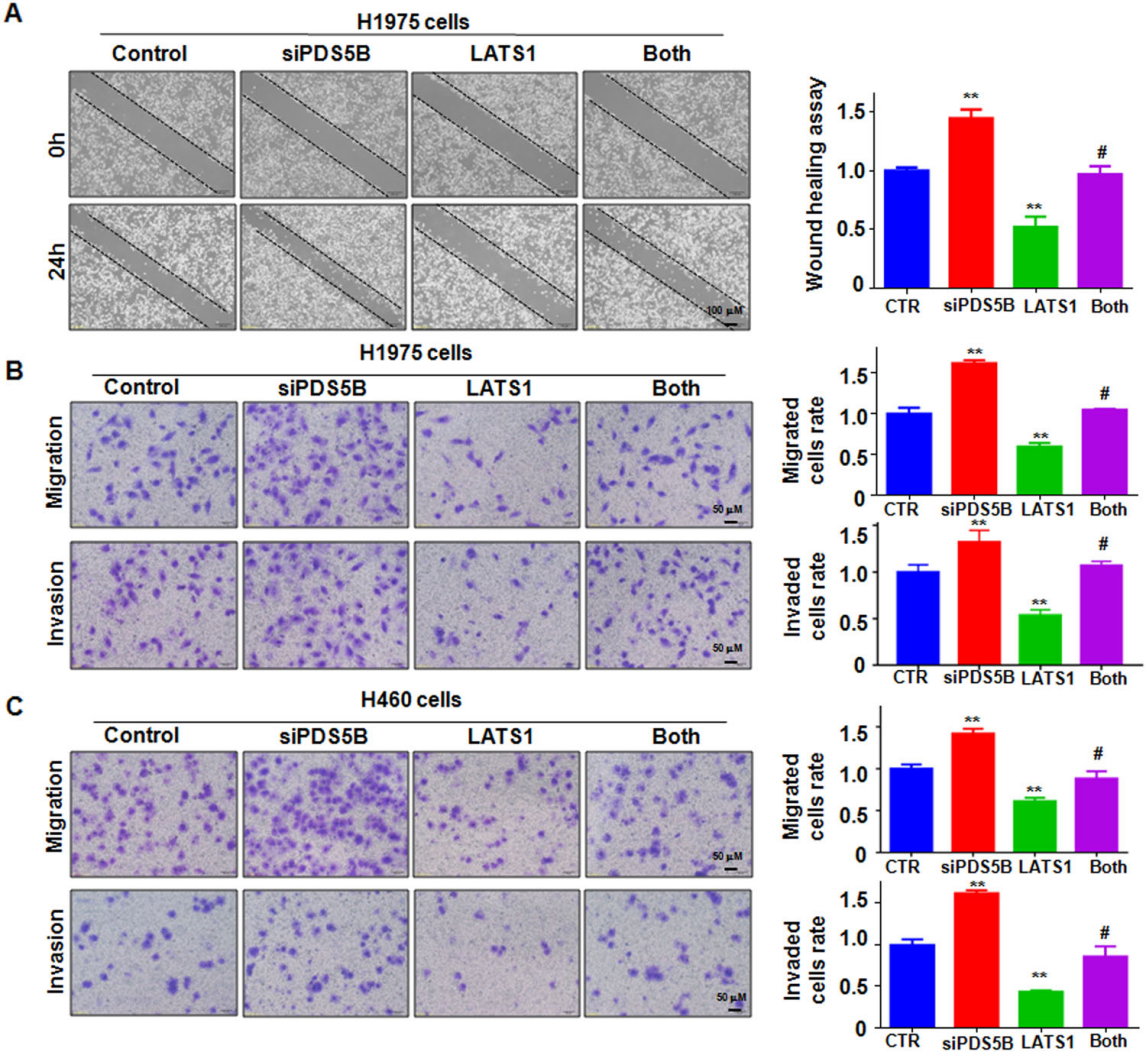
LATS1 overexpression abrogates PDS5B knockdown-mediated motility. **A** Left panel: wound closure was examined by wound-healing assays in H1975 cells after co-transfection of LATS1 cDNA and PDS5B siRNA. Right panel: quantitative data of wound closure. **P < 0.05 vs control group; ^#^*P* < 0.05 vs PDS5B siRNA alone or LATS1 cDNA alone. *Control* nonspecific control siRNA and empty vector, *siPDS5B* PDS5B siRNA, *LATS1* LATS1 cDNA, *Both* siPDS5B + LATS1. **B** Left panel: cell motility was examined in H1975 cells after co-transfection of LATS1 cDNA and PDS5B siRNA. Right panel: quantitative data of cell motility. **P < 0.05 vs control group; ^#^*P* < 0.05 vs PDS5B siRNA alone or LATS1 cDNA alone. **C** Left panel: cell motility was measured in H460 cells after co-transfection of LATS1 cDNA and PDS5B siRNA. Right panel: Quantitative data of cell motility. **P < 0.05 vs control group; ^#^*P* < 0.05 vs PDS5B siRNA alone or LATS1 cDNA alone. Data are shown as mean ± SD from experiments in triplicate.

Next, we performed wound-healing assays in NSCLC cells after PDS5B knockdown in combination with LATS1 overexpression. Upregulation of LATS1 delayed wound closure in H1975 and H460 cells (Fig. 5A). Moreover, overexpression of LATS1 rescued PDS5B siRNA-mediated enhancement of wound closures in H1975 and H460 cells (Fig. 5A). Furthermore, LATS1 upregulation suppressed migration and invasion of H1975 and H460 cells by transwell chamber assays (Fig. 5B). Strikingly, overexpression of LATS1 abolished PDS5B silencing-induced migration and invasion in two NSCLC cell lines (Fig. 5B). Thus, PDS5B inhibited cell motility partly via upregulation of LATS1 in NSCLC cells.

### Overexpression of PDS5B suppresses tumor growth in vivo

**Fig. 6:**
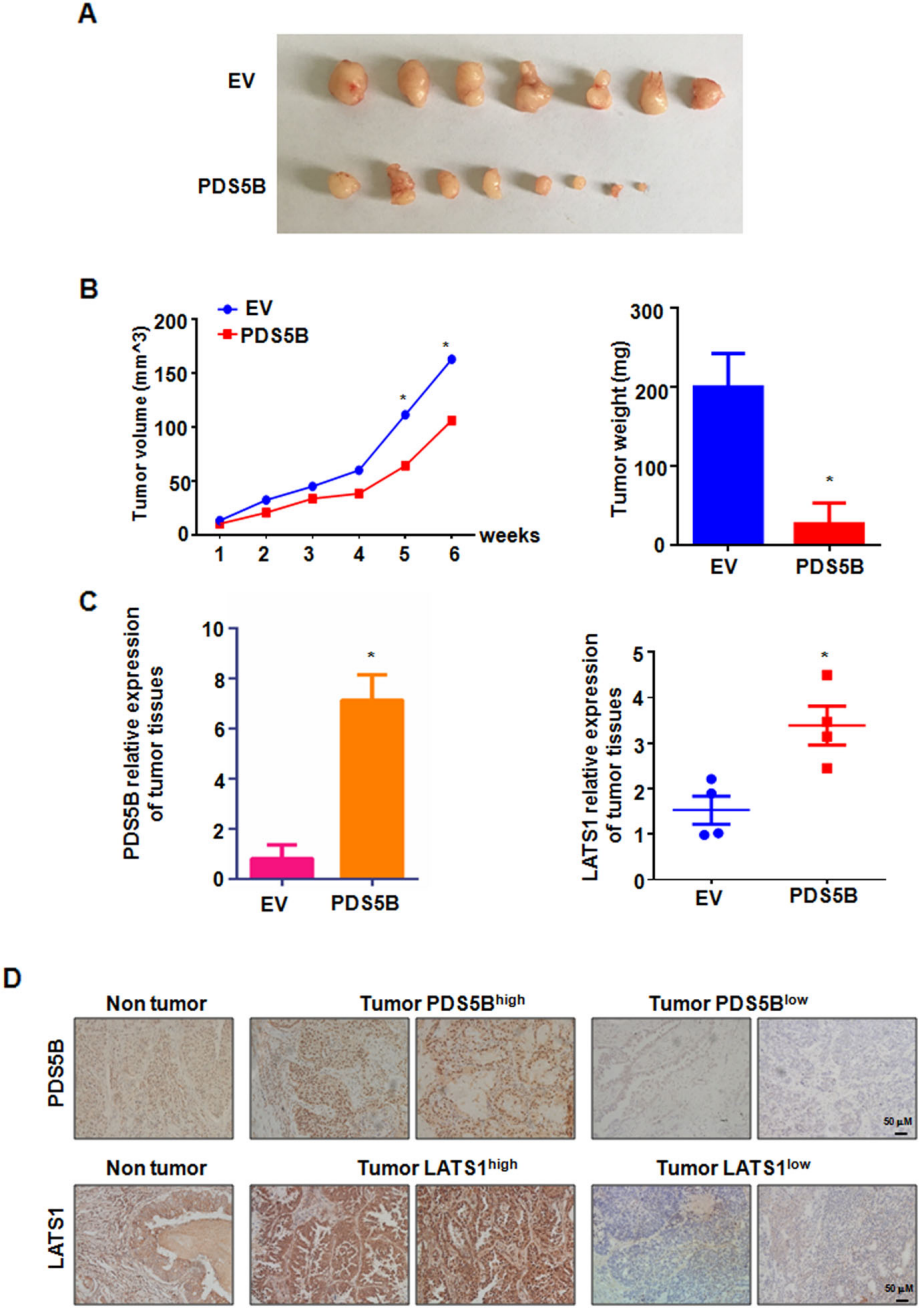
Overexpression of PDS5B suppresses tumor growth in vivo. **A**. The tumors in nude mice were dissected and taken a picture. *EV* empty vector, *PDS5B* PDS5B cDNA. EV group is the control group. **B** In vivo tumor growth was measured per week and the weights of the dissected tumors. Data are shown as mean ± SD. *P < 0.05 vs EV group. **C** The expression of PDS5B and LATS1 in tissues from xenografted tumors. Data are shown as mean ± SD. *P < 0.05 vs EV group. **D** Immunohistochemical staining images of PDS5B and LATS1 in lung cancer tissues.

To determine whether PDS5B could retard tumor growth in vivo, we performed a mouse study using lung cancer xenografts in nude mice. We found that PDS5B overexpression reduced tumor growth compared with the control group (Fig. 6A, B). The tumor weights and volumes were less in the PDS5B-overexpressing group (Fig. 6A, B). Furthermore, the expression of PDS5B and LATS1 was determined in mouse tumor tissues by western blotting. We observed that LATS1 expression was increased in the PDS5B-overexpressing group (Fig. 6C).

### PDS5B expression is associated with lymph node metastasis

To ascertain the expression of PDS5B in tumor specimens of lung cancer patients, we used immunohistochemical staining to detect PDS5B expression levels in tumor tissues of 232 patients with lung cancer. PDS5B was detected to be expressed in 39 tumor tissues, and 193 patients displayed no detectable expression of PDS5B. Moreover, low expression of PDS5B was associated with lymph node metastasis in lung cancer patients. In addition, 70% of lung cancer patients exhibited undetectable or lower expression of LATS1. Notably, the expression of PDS5B was correlated with LATS1 expression levels in tumor specimens in lung cancer patients (Fig. 6D).

## Discussion

PDS5B is an important factor for proper cohesion dynamics and protection of replication fork [23]. The Shu complex, which consists of SWS1 and SWSAP1, interacts with PDS5B and SPIDR and governs DNA repair [24]. PDS5B has been reported to involve in tumorigenesis and tumor progression. For instance, low expression of PDS5B is associated with better survival in patients with ovarian cancer [25]. In the current study, low expression of PDS5B was observed and correlated with lymph node metastasis in lung cancer patients, indicating that PDS5B might be associated with the prognosis of lung cancer patients. One study revealed that PDS5B exerts antitumor activity in pancreatic cancer cells [26]. Overexpression of PDS5B represses cell viability and stimulates apoptosis in pancreatic cancer cells, while depletion of PDS5B increases migratory and invasive ability [26]. In support of the tumor-suppressive role of PDS5B, our present study demonstrated that PDS5B upregulation repressed viability, migration, and invasion of lung cancer cells, while PDS5B knockdown promoted viability and motility of NSCLC cells. Strikingly, PDS5B upregulation retarded tumor growth in vivo, further supporting the anticancer role of PDS5B in lung cancer.

PDS5B alleviates cell viability, migration, and invasion via promoting Ptch2 expression in pancreatic cancer cells [27]. Interestingly, one group reported that PD-L1 competes with WAPL to bind to PDS5B and performs tumor progression independent of its role in the immune checkpoint [28]. Moreover, silencing of PDS5B expression promotes cell proliferation via induction of IL-6 secretion and activation of STAT3, and promotion of cyclin D1 expression in human cancer [29]. In the present study, we reported that PDS5B positively regulated the expression of LATS1 in lung cancer cells. Notably, overexpression of LATS1 abrogated PDS5B knockdown-induced tumor promotion in NSCLC cells, suggesting that LATS1 is involved in PDS5B-mediated anticancer activity in lung cancer. In line with this concept, PDS5B was associated with the expression of LATS1 in tumor tissues of lung cancer patients.

It has been reported that miR-223 promotes the viability and motility of pancreatic cancer cells via inhibition of PDS5B in pancreatic cancer cells [26]. It is required to investigate whether miR-223 or other miRNAs target PDS5B expression in lung cancer cells, which will help us understand the regulatory mechanism of PDS5B. Downregulation of PDS5B increased the sensitivity of the PARP inhibitor Olaparib, indicating that PDS5B is a potential target for treating ovarian cancer [25]. It is necessary to determine whether upregulation of PDS5B could enhance the sensitivity of chemotherapeutic drugs in lung cancer. As PDS5B might be a useful target for fighting cancer, it is pivotal to discover the compounds to modulate the expression of PDS5B and to obtain a better treatment outcome for cancer patients.

## Materials and methods

### Cell culture

Human lung epithelial BEAS-2B cells were kindly provided by EK-Bioscience Company (Shanghai, China). Human non-small-cell lung cancer cell lines (H1975 and H460 cells) were cultured in Roswell Park Memorial Institute Medium (RPMI)-1640 medium, supplemented with 10% fetal bovine serum (FBS, Gibco, USA) and 1% streptomycin and penicillin in a humidified atmosphere containing 5% CO_2_ at 37 °C.

### Transfection

The H1975 and H460 cells were grown in six-well plates and transfected with PDS5B cDNA or PDS5B siRNA or nonspecific control siRNA or empty vector using Lipofectamine 3000 according to manufacturer’s protocol [30]. The PDS5B siRNAs were purchased from GenePharma Company (Shanghai, China).

### MTT assays

The H1975 and H460 cells were grown in 96-well plates. After overnight incubation, NSLC cells were transfected with PDS5B cDNA or PDS5B siRNA for 24, 48, and 72 h. Then, MTT assay was performed as described before [31]. In brief, the transfected cells were harvested and MTT agent (50 μL) was incubated to each well for 2 h. Dimethyl sulfoxide was used to dissolve formazan cells after the supernatant was removed. The OD value at 590 nm was measured by a microplate reader for detecting cell viability.

### Wound-healing assays

The transfected lung cancer cells were grown in six-well plates and incubated at 37 °C. When the cells obtained 90% of confluence, the wound scrape was created by a 100 μl sterile pipette tip. The supernatant cells were washed away with PBS. The scratched areas were imaged at 0 h and 24 h, respectively [31]. The distances between gaps were calculated.

### Transwell migration and invasion assays

The 24-well plate with Transwell chambers was utilized for detecting migration and invasion. The transfected cells with RPMI-1640 medium without FBS were seeded into the upper inserts. RPMI-1640 medium with FBS was added into the lower chamber. Non-migrated cells or non-invaded cells were removed by cotton swabs after 24 h. The migrated and invaded cells were stained with Crystal Violet. The images were captured by a microscope as described before [32].

### Western blotting analysis

H1975 and H460 cells were harvested after PDS5B modulation and total proteins from cells were extracted. The expression of PDS5B and LATS1 was measured by Western blotting analysis as described previously [31]. The protein from cell lysate was separated by sodium dodecyl-sulfate polyacrylamide gel electrophoresis and transferred to the polyvinylidene difluoride membrane. Then, the membranes were incubated with anti-PDS5B, anti-LATS1, or anti-tubulin antibodies. After washed by TBST three times, the membranes were incubated with the secondary antibodies for 1 h at room temperatures. The anti-PDS5B antibody was obtained from Bethyl laboratories. Anti-PDS5B antibody, anti-tubulin antibody, and the secondary antibodies were bought from Cell Signaling Technology (Danvers, MA, USA). The bands were visualized by the Tanon5200 (Shanghai, China) and ImageJ software was used to measure the intensity of proteins.

### In vivo experiments

The animal study was approved by the Animal Care and Use Committee of Bengbu Medical College. The in vivo experiments were operated following the previous description [33]. To establish the lung cancer xenografts, the H1975 cells with overexpressed PDS5B or the control cells transfected with empty vector were injected into five-week-old male nude mice (8 mice/group). After 2 weeks of injection, the tumor size was measured with a caliper per week. The weights of tumor masses were detected after the mice were executed at 6 weeks.

### Human lung cancer patient samples

The tumor samples of lung cancer patients and their matched adjacent non-cancerous tissues were obtained from Outdo Biotech Company (Shanghai, China). The expression of PDS5B and LATS1 was determined by immunohistochemical studies in tumor specimens [27].

### Statistical analysis

All results were presented as the mean ± standard deviation. All data are representative of three independent experiments. A Student’s *t* test was used to evaluate the differences between the two groups. Analysis of variance followed by Tukey’s post hoc test was used to compare the differences among multiple groups. *P* < 0.05 was considered statistically significant.
